# *Acinetobacter geminorum* sp. nov., isolated from human throat swabs

**DOI:** 10.1099/ijsem.0.005018

**Published:** 2021-10-11

**Authors:** Sophia Wolf, Elisabeth Barth-Jakschic, Karolin Birkle, Baris Bader, Matthias Marschal, Jan Liese, Silke Peter, Philipp Oberhettinger

**Affiliations:** ^1^​ Institute of Medical Microbiology and Hygiene, University of Tuebingen, Tuebingen, Germany; ^2^​ German Center for Infection Research (DZIF), partner site Tuebingen, Tuebingen, Germany

**Keywords:** *Acinetobacter calcoaceticus–baumannii* complex, new species, Next-generation sequencing, phylogeny, average nucleotide identity

## Abstract

Two isolates of a non-fermenting, Gram-negative bacterial strain were cultured from two throat swabs that were taken from a pair of twins during routine microbiological surveillance screening. As these isolates could not be unambiguously identified using routine diagnostic methods, whole genome sequencing was performed followed by phylogenetic analysis based on the *rpoB* gene sequence and by whole genome datasets. The two strains compose a separate branch within the clade formed by the *Acinetobacter calcoaceticus–baumannii* (ACB) complex with *Acinetobacter pittii* CIP 70.29^T^ as the most closely related species. The average nucleotide identity compared to all other species of the ACB complex was below 94.2% and digital DNA–DNA hybridization values were less than 60%. Biochemical characteristics confirm affiliation to the ACB complex with some specific phenotypic differences. As a result of the described data, a new *Acinetobacter* species is introduced, for which the name *Acinetobacter geminorum* sp. nov. is proposed. The type strain is J00019^T^ with a G+C DNA content of 38.8 mol% and it is deposited in the DSMZ Germany (DSM 111094^T^) and CCUG Sweden (CCUG 74625^T^).

## Isolation and ecology

Most *Acinetobacter* species are environmental organisms found in soil and wetlands [1], usually non-pathogenic for humans and frequently isolated as coloniser of skin among healthy patients [2, 3]. However there are also clinically relevant *Acinetobacter* species causing mainly hospital-acquired infections mostly in intensive-care settings [4]. These are varying from urinary tract [5] and soft tissue infections [6] to pneumonia [7], endocarditis [8] and bacteraemia [9] with more than 1 million cases per year worldwide [10, 11] together with reduction of antimicrobial susceptibility [12]. These species form a phylogenetically defined clade of phenotypically and genomically related species [13–16] referred to as the *Acinetobacter calcoaceticus–baumannii* (ACB) complex comprising *Acinetobacter calcoaceticus *, *Acinetobacter baumannii *, *Acinetobacter nosocomialis* and *Acinetobacter pittii* [17–21], in which *A. calcoaceticus* is not considered clinically relevant and resistance against antibiotics is unusual.

In 1911, Beijernick *et al*. described the species *Micrococcus calcoaceticus* as the first reference for an *Acinetobacter* species [22]. In 1954, the new genus was introduced into taxonomy by Brisou and Prévot with the revision of different species summarized into the genus *Achromobacter* so far [23]. Finally, in 1968, Baumann *et al*. reclassified several genera and species into the genus *Acinetobacter* and presented a modified description of the genus [24]. At the time of writing, 65 validly published *Acinetobacter* species were described (www.bacterio.net/acinetobacter.html) supplemented by 13 additional species that were non-validly published.

In the last decade, three more *Acinetobacter* species were described clustering into the ACB complex: *Acinetobacter oleivorans* [25], *Acinetobacter seifertii* [16] and *Acinetobacter lactucae* [26]. In the present study, two isolates of a new *Acinetobacter* species were identified in the context of the weekly screening of a neonatology ward corresponding to the guidelines of the German Committee of Hospital Hygiene and Infection Prevention of the Robert Koch Institute [27]. These isolates were analysed by whole genome sequencing (WGS) and genomic data were used for calculation of phylogeny, determination of nucleotide diversity and biochemical characterization in relation to available datasets of validly and effectively published *Acinetobacter* species (apps.szu.cz/anemec/Classification.pdf). The presented results indicate that the two new *Acinetobacter* isolates should be classified as a new member of the ACB complex.

## Methods

Microbiological screening of all patients is routinely performed on the neonatal intensive care unit of our university hospital. Two throat swabs from a pair of twins yielded two isolates that belonged to the genus *Acinetobacter *. These isolates could not be unambiguously identified using a MALDI-TOF Microflex LT instrument (Bruker Daltonics; MBT IVD Library.5627) or the VITEK 2 GN identification system (bioMérieux) [28]. The best hit generated by the Microflex LT system was for the *A. baumannii* complex with a MALDI-TOF score value below 2.0. For a clear identification at the species level, a MALDI-TOF score >2.0 is expected and a delimitation of the best match is ensured by a score >0.3 higher compared to the next species.

Bacterial DNA was extracted from cultures grown on Columbia agar with 5% sheep blood (Becton Dickinson) using the DNeasy UltraClean Microbial Kit (Qiagen) following the manufacturer’s instructions with some minor modifications. Libraries for WGS were prepared using the TruSeq DNA HT Sample Prep Kit (Illumina) with 96 different barcodes using standard protocols as described previously [29–31]. Normalized libraries were pooled and sequenced with a Mid Output Kit version 2.5 (2×150 bp) on a NextSeq platform (Illumina). Genomic sequencing reads were assembled using SPAdes (version 3.7.0) [32] with default settings.

WGS data of publicly available *Acinetobacter* type strains (summarized in Table S1, available in the online version of this article) were used for phylogenetic analysis of the two *Acinetobacter* isolates. ProgressiveMauve (version 2.3.1) [33] using default settings was run to conduct a multi-fasta alignment of 61 *Acinetobacter* genomes including the two new *Acinetobacter* isolates, representing all published *Acinetobacter* species up to the time of writing. Prophage regions were investigated using phaster (phaster.ca) [34]. The multi-fasta alignment was used to reconstruct a maximum-likelihood phylogenetic tree of all 61 *Acinetobacter* species isolates applying iq-tree with 1000 bootstrap replicates using the UFboot algorithm. *rpoB* (β-subunit of RNA polymerase) sequences, used for a recent description of new *Acinetobacter* species as the most reliable method for species delineation [35], were extracted from available WGS data and aligned with ClustalW (BioEdit version 7.2.5) followed by phylogenetic treeing with RAxML and the GTR model in conjunction with gamma rates [36]. Visualization of trees was done using FigTree (version 1.4.3). The average nucleotide identity (ANI) of selected *Acinetobacter* type strains was assessed by JSpecies (version 1.2) [37, 38] based on blast+ 2.20.29 (ANIb). The Genome-to-Genome Distance Calculator (GGDC 2.1) using the recommended Formula 2 was applied for *in silico* genome comparison and computation of digital DNA–DNA hybridization (dDDH) values [39].

Assimiliation and temperature growth tests of the new *Acinetobacter* isolates were performed using the standard panel for *Acinetobacter* species (www.szu.cz/anemec/Phenotype.pdf) by the Laboratory of Bacterial Genetics, National Institute of Public Health, Prague, Czech Republic, as described previously [40–42].

Motility assays were performed as described earlier [43] and were performed in three independent experiments.

## Phylogeny

**Fig. 1.**
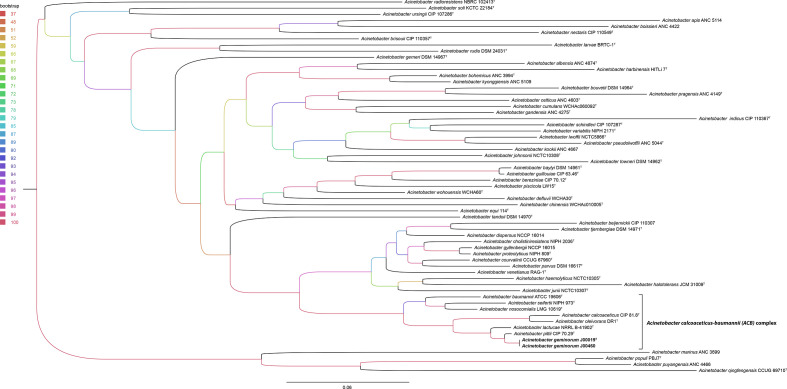
Phylogeny based on extracted *rpoB* sequences of the study isolates (J00019^T^, J00460) and available genome data of *Acinetobacter* type strains. The scale bar represents the expected number of changes per site. Bootstrap values (%) are colour-coded for all nodes (based on 1000 replicates). The tree is rooted at the midpoint. The ACB complex is highlighted and the two study isolates are labelled in bold.

**Fig. 2.**
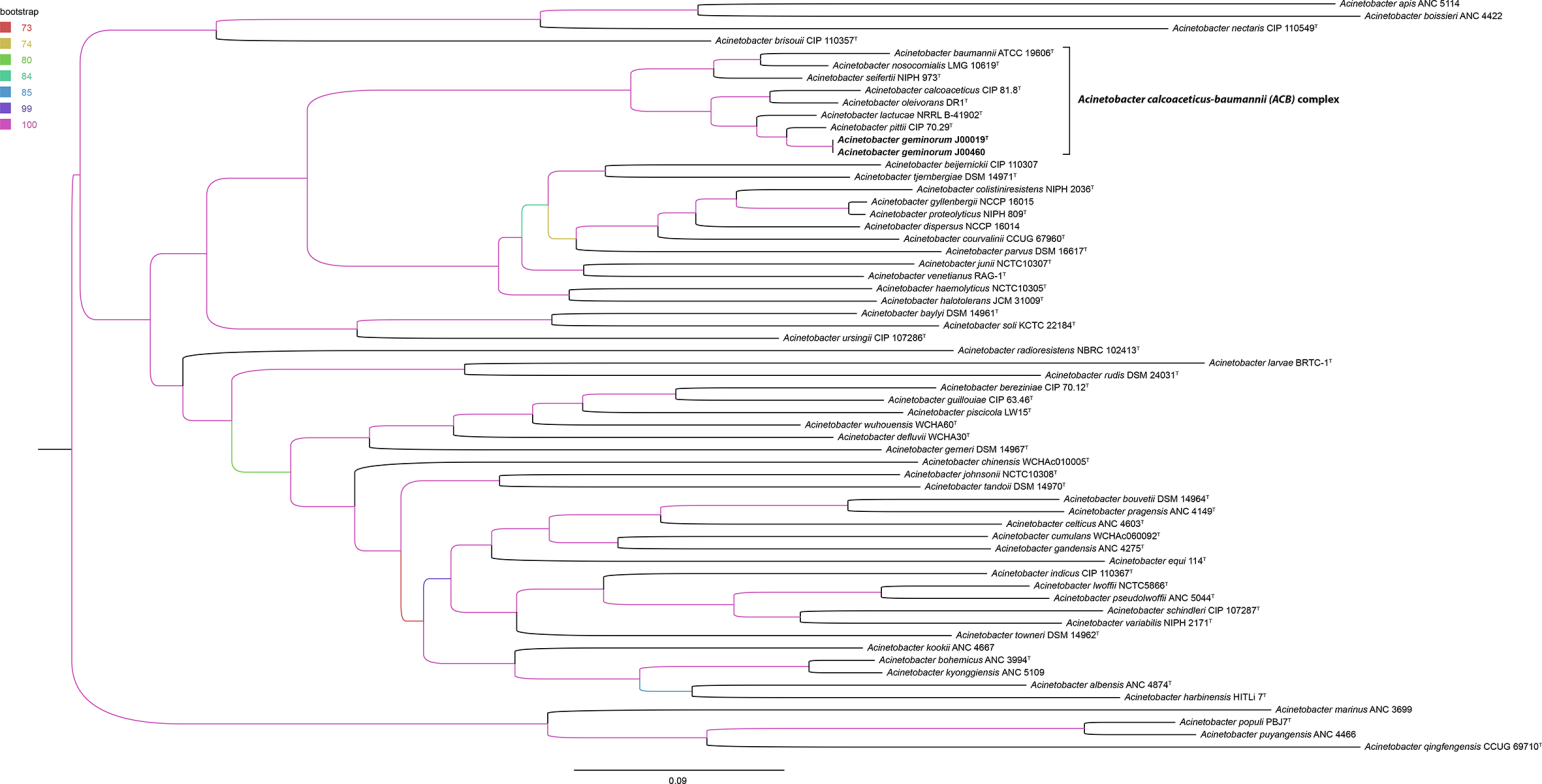
Maximum-likelihood phylogeny based on multi-fasta alignment of whole genome sequencing data of the study isolates (J00019^T^, J00460) and available genome data of *Acinetobacter* type strains. The scale bar represents the expected number of changes per site. Bootstrap values (%) are colour-coded for all nodes (based on 1000 replicates). The tree is rooted at the midpoint. The ACB complex is highlighted and the two study isolates are labelled in bold.

The phylogenetic relationship of the two *Acinetobacter* study isolates was analysed in relation to other available *Acinetobacter* type strains using WGS data. The 16S rRNA gene has only a limited polymorphism for discrimination of *Acinetobacter* species [44, 45]. Comparing full-length 16S rRNA gene similarity of all type strains of the ACB complex, the sequence identity was between 99.11 and 99.93%, representing the low discrimination limit of ACB complex members. Only *A. baumannii* ATCC 19606^T^ can be clearly separated from J00019^T^ using 16S rRNA gene analysis with an identity of 97.33%. Therefore phylogenetic analysis was conducted based on the full-length housekeeping-gene *rpoB* (Fig. 1) independently extracted from WGS data of type strains. The *rpoB* gene, which is one of the best-studied single-gene phylogenetic markers, was widely used in recently published nomenclature proposals for taxonomic status of the genus *Acinetobacter* [46, 47]. The maximum-likelihood phylogeny based on *rpoB* shows clustering of the two isolates J00019^T^ and J00460 together in a separate branch distinct from *A. pittii* CIP 70.29^T^, the most closely related type species. Interestingly, the two new *Acinetobacter* isolates belong to a distinct clade including all species of the ACB complex, a subgroup of strains with high clinical relevance [48]. In addition to *rpoB*-based phylogeny, a phylogenetic tree based on multi-fasta alignment of WGS data enabled further distinction of the new *Acinetobacter* species from other type strains of the genus including the most closely related species *A. pittii* CIP 70.29^T^ and all other *Acinetobacter* species of the ACB complex (Fig. 2).

## Genome features

Delineation of new bacterial species can be done using genomic sequencing data. Therefore, the ANI value [37, 49] as well as the recently described dDDH value can be used for description of a new bacterial species [50]. The new *Acinetobacter* isolates were compared to a subset of closely related *Acinetobacter* type strains selected due to clustering in the phylogenetic tree. The closest relationship of the new *Acinetobacter* species below the proposed cut-off value of 95–96% for the assignment of a new species [50] was found to *A. pittii* CIP 70.29^T^ (94.18%), the direct neighbour in the phylogenetic tree. In comparison, the ANI value between the two study isolates (J00019^T^ and J00460) was 99.98%, demonstrating their close relationship (Table 1).

**Table 1.** Percentage of average nucleotide identity (ANI) and calculation of digital DNA–DNA-hybridization (dDDH) for the two study isolates and closely related *Acinetobacter* type strains

		1		2		3		4		5		6		7		8		9	
		ANI	dDDH	ANI	dDDH	ANI	dDDH	ANI	dDDH	ANI	dDDH	ANI	dDDH	ANI	dDDH	ANI	dDDH	ANI	dDDH
**1**	J00019^T^	*	*	99.98	100.00	94.18	59.20	92.47	49.00	90.53	42.00	89.43	39.30	87.29	34.00	87.11	32.80	87.40	34.00
**2**	J00460	99.98	100.00	*	*	94.17	58.20	92.48	49.00	90.47	42.00	89.33	39.30	87.25	34.00	87.10	32.80	87.35	34.00
**3**	* Acinetobacter pittii* CIP70.29^T^	93.87	59.20	93.86	59.20	*	*	93.09	51.20	90.69	43.10	89.35	38.90	87.34	34.50	87.24	33.40	87.63	34.90
**4**	* Acinetobacter lactucae* NRRL B-41902^T^	92.24	49.00	92.25	49.00	93.09	51.20	*	*	90.56	42.90	89.42	39.00	86.93	33.40	86.75	32.40	87.17	33.80
**5**	* Acinetobacter oleivorans* DR1^T^	90.15	42.00	90.16	42.00	90.54	43.10	90.53	42.90	*	*	91.17	44.90	87.45	34.50	86.84	33.00	86.79	33.00
**6**	* Acinetobacter calcoaceticus* CIP 81.8^T^	89.36	39.30	89.36	39.30	89.41	38.90	89.50	39.00	91.34	44.90	*	*	86.09	31.60	86.14	31.50	86.22	31.60
**7**	* Acinetobacter seifertii* NIPH 973^T^	87.24	34.00	87.22	34.00	87.24	34.50	86.86	33.40	87.41	34.50	85.87	31.60	*	*	91.73	46.80	89.26	39.30
**8**	* Acinetobacter nosocomialis* LMG 10619^T^	87.11	32.80	87.10	32.80	87.32	33.40	86.84	32.40	86.95	33.00	86.06	31.50	91.77	46.80	*	*	91.02	44.60
**9**	* Acinetobacter baumannii* ATCC 19606^T^	87.20	34.00	87.21	34.00	87.64	34.90	87.25	33.80	86.90	33.00	86.22	31.60	89.38	39.30	91.10	44.60	*	*

*n/a

In addition, dDDH values were calculated for the selected subset of *Acinetobacter* type strains in relation to the new *Acinetobacter* species. The lowest intergenomic distance of the two analysed *Acinetobacter* species isolates was also found to *A. pittii* CIP 70.29^T^ with a dDDH value of 59.20%, clearly below the cutoff proposed of 70% for bacterial species delineation [37, 39, 49, 50]. The dDDH value between the novel *Acinetobacter* isolates was 100%.

## Physiology

Phenotypic discrimination of the different members of the ACB complex is challenging as described previously [20, 21]. Biochemical profiling of the new *Acinetobacter* isolates obtained via assimiliation and temperature growth tests was kindly performed in the lab of A. Nemec in Prague using the standard panel for *Acinetobacter* species (www.szu.cz/anemec/Phenotype.pdf) and compared with the six species of the ACB complex with validly published names. The data are concordant with published data [16, 21, 25, 26] of the ACB complex and closely related strains (Table 2). The novel *Acinetobacter* species has some unusual characteristics as it is not able to grow with adipate, azelate and 4-hydroxybenzoate. The inability to assimilate these three substances separate the novel *Acinetobacter* species from the closely related *A. pittii *. Additionally, J00019^T^ and J00460 were negative for the assimilation of l-arabinose, l-leucine, l-phenylalanine and phenylacetate, whereas most *A.pittii* isolates as well as *A.dijkshoorniae*/*A.lactucae* were positive in this test. The inability to assimilate l-phenylalanine and phenylacetate can also be used to separate the two novel *Acinetobacter* isolates from *A. calcoaceticus.* Strain J00019^T^ can be distinguished from *A. baumannii* by being negative for glutamyl-arylamidase pNA and positive for tyrosine arylamidase. The swarming inability of strain J00019^T^ on motility agar plates allowed phenotypic discrimination of the new species from *A. oleivorans* (data not shown).

**Table 2.** Biochemical characteristics of the two *Acinetobacter geminorum* study isolates and closely related *Acinetobacter* species clustering in the ACB complex Numbers in the table are percentages of strains with clearly positive reactions and results for type strains are given in parentheses. D means that the reactions were mostly doubtful or irreproducible, + or – stand for positive or negative for the respective reaction. The number of strains tested is indicated next to the respective species name in parentheses. Data and a slightly modified table were kindly provided by A. Nemec.

Characteristic	J00019^T^, J0046	* A. pittii* (20)	* A. dijkshoorniae */ * A. lactucae* (6)	* A. calcoaceticus* (11)	* A. nosocomialis* (20)	* A. seifertii* (16)	* A. baumannii* (25)
Growth at 44 °C	–	10 (–)	50 (+)	–	95 (+)	13 (–)	+
Growth at 41 °C	+	+	+	9 (–)	+	94 (+)	+
Growth at 37 °C	+	+	+	91 (–)	+	+	+
Growth at 35 °C	+	+	+	+	+	+	+
Growth at 32 °C	+	+	+	+	+	+	+
Acidification of d-glucose	+	95 (+)	+	91 (–)	+	+	+
Haemolysis of sheep blood	–	–	–	–	–	–	–
Liquefaction of gelatin	–	–	–	–	–	–	–
Assimilation of:							
Acetate	+	+	+	+	+	+	+
trans-Aconitate	+	+	+	+	60 (+)	+	92 (+)
Adipate	–	+	+	+	95 (+)	63 (–)	88 (+)
β-Alanine	+	90 (+)	+	91 (–)	85 (+)	88 (–)	+
4-Aminobutyrate	+	+	+	+	+	+	+
l-Arabinose	–	85 (+)	+	27 (–)	+	–	84 (+)
l-Arginine	+	+	+	+	+	+	+
l-Aspartate	+	+	+	+	+	+	+
Azelate	–	+	+	+	95 (+)	63 (–)	88 (+)
Benzoate	+	90 (+)	+	+	90 (+)	94 (+)	84 (+)
2,3-Butanediol	+	85 (+)	+	+	90 (+)	+	+
Citraconate	–	–	–	–	–	–	40 (+)
Citrate (Simmons)	+	+	+	91 (D)	+	+	+
Ethanol	+	+	+	91 (+)	+	+	96 (+)
Gentisate	–	25 (–)	+	–	10 (–)	75 (+)	4 (–)
d-Gluconate	–	–	–	–	–	–	–
d-Glucose	–	–	–	–	–	–	–
l-Glutamate	+	+	+	+	+	+	+
Glutarate	+	90 (+)	+	91 (D)	95 (+)	+	96 (+)
Histamine	–	–	–	–	–	–	–
l-Histidine	+	+	+	+	+	94 (+)	96 (+)
4-Hydroxybenzoate	–	+	+	91 (+)	80 (+)	94 (+)	92 (+)
dl-Lactate	+	+	+	+	+	+	+
l-Leucine	–	95 (+)	+	91 (–)	95 (+)	94 (–)	88 (+)
Levulinate	–	5 (–)	67 (+)	91 (–)	5 (–)	6 (–)	24 (-)
d-Malate	+	95 (+)	+	D (-)	+	88 (+)	92 (+)
Malonate	+	95 (+)	+	+	20 (+)	75 (+)	88 (+)
l-Ornithine	+	95 (+)	+	+	95 (+)	81 (–)	76 (–)
Phenylacetate	–	75 (+)	+	+	85 (+)	88 (+)	84 (+)
l-Phenylalanine	–	75 (+)	+	+	85 (+)	88 (+)	84 (+)
Putrescine	+	+	+	+	95 (+)	81 (–)	96 (+)
d-Ribose	–	35 (–)	83 (+)	45 (–)	80 (+)	–	76 (+)
l-Tartrate	–	85 (+)	–	9 (–)	–	31 (+)	32 (–)
Tricarballylate	+	+	+	+	95 (+)	+	92 (+)
Trigonelline	–	20 (+)	67 (+)	9 (–)	20 (–)	–	60 (+)
Tryptamine	–	–	50 (+)	–	–	6 (–)	–

Detailed MALDI-TOF analysis using a Microflex LT instrument (Bruker Daltonics; MBT IVD Library.5672) for strain J00019^T^, strain J00460 and *A. pittii* DSM25618^T^ performed in quadruplicate resulted in specific peaks for the two *Acinetobacter* species with high masses. This indicates that strains J00019^T^ and J00460 belong to a different species compared to the most closely related *A. pittii* type strain as well as to 17 verified *A. pittii* isolates included in our MALDI database (data not shown).

Taken together, phylogenetic analysis based on *rpoB* and WGS data, calculation of genome relatedness by ANI and dDDH as well as biochemical properties classifies strains J00019^T^ and J000460 as a new species within the genus *Acinetobacter* for which we propose the name *Acinetobacter geminorum* sp. nov. with J00019^T^ as the type strain.

## Description of *Acinetobacter geminorum* sp. nov.

*Acinetobacter geminorum* (ge.mi.no'rum. L. gen. pl. n. *geminorum*, pertaining to the twins from which the two isolates were collected).

Gram-stain-negative, oxidase-negative, catalase-positive, non-fermenting, non-motile, strictly aerobic, rod-shaped bacterium. The colonies are grey, slightly shiny, convex and circular with 1 mm in diameter after 24 h of growth on blood agar plates. Temperature from 37 to 41 °C is tolerated and no haemolysis is observed.

The physiological profiles of the two strains are congruent with those of the ACB complex. The strains are able to assimilate acetate, citrate (Simmons), l-glutamate, glutarate, l-histidine, dl-lactate, d-malate, malonate and l-ornithine. In contrast to most of the ACB complex strains, J00019^T^ and J00460 are negative for assimilation of adipate, l-arabinose, azelate, 4-hydroxybenzoate, l-leucine, phenylacetate and l-phenylalanine.

The type strain of *Acinetobacter geminorum* is J00019^T^, isolated from a throat swab of a patient hospitalized at the University Hospital Tuebingen, Germany. The G+C DNA content of the type strain is 38.8 mol%. The culture certificate accession numbers are CCUG 74625^T^ from the CCUG, Göteborg, Sweden, and DSM 111094^T^ from the DSMZ, Braunschweig, Germany.

## Supplementary Data

Supplementary material 1
