# Supplementary material for: Acinetobacter geminorum sp. nov., isolated from human throat swabs
Source: Int J Syst Evol Microbiol. 2021 Oct 11;71(10):005018. doi: 10.1099/ijsem.0.005018 (PMC8604166; doi:10.1099/ijsem.0.005018)
Supplement: Supplementary material 1 [file ijsem-71-5018-s001.pdf]

Supplementary Figures and Tables

Supplementary Table S1: Overview of *Acinetobacter* species type strains, strain identification and corresponding nucleotide accession/Gen Bank numbers of the strains included in the study.

	Whole genome sequencing data	
***Acinetobacter* species**	**Strain ID**	**Gen Bank number**
Acinetobacter albensis	ANC 4874^{T}	NZ_FMBK00000000.1
Acinetobacter apis	ANC 5114	NZ_FZLN00000000.1
Acinetobacter baumannii	ATCC 19606^{T}	NZ_CP046654.1
Acinetobacter baylyi	DSM 14961^{T}	NZ_JHZI00000000
Acinetobacter beijernickii	CIP 110307	NZ_APQL00000000.1
Acinetobacter bereziniae	CIP 70.12^{T}	NZ_APQG00000000.1
Acinetobacter bohemicus	ANC 3994^{T}	NZ_KB849163.1
Acinetobacter boissieri	ANC 4422	NZ_FMYL00000000.1
Acinetobacter bouvetii	DSM 14964^{T}	NZ_KB849725.1
Acinetobacter brisouii	CIP 110357^{T}	NZ_KI530762.1
Acinetobacter calcoaceticus	CIP 81.8^{T}	NZ_KB849778.1
Acinetobacter celticus	ANC 4603^{T}	NZ_MBDL00000000.1
Acinetobacter chinensis	WCHAc010005^{T}	NZ_CP032134.1
Acinetobacter colistiniresistens	NIPH 2036^{T}	NZ_KE340374.1
Acinetobacter courvalinii	CCUG 67960^{T}	NZ_VZOE00000000.1
Acinetobacter cumulans	WCHAc060092^{T}	NZ_CP035934.2
Acinetobacter defluvii	WCHA30^{T}	NZ_CP029397.2
Acinetobacter dispersus	NCCP 16014	NZ_CP041970.1
Acinetobacter equi	114^{T}	NZ_CP012808.1
Acinetobacter gandensis	ANC 4275^{T}	NZ_LZDS01000001.1
Acinetobacter gerneri	DSM 14967^{T}	NZ_KB849514.1
Acinetobacter guillouiae	CIP 63.46^{T}	NZ_KB849280.1
Acinetobacter gyllenbergii	NCCP 16015	NZ_CP041971.1
Acinetobacter haemolyticus	NCTC10305^{T}	NZ_UFRR01000003.1
Acinetobacter halotolerans	JCM 31009^{T}	NZ_SGIM00000000.1
Acinetobacter harbinensis	HITLi 7^{T}	NZ_JXBK01000001.1
Acinetobacter indicus	CIP 110367^{T}	NZ_KI530745.1
Acinetobacter junii	NCTC10307^{T}	NZ_UFRZ01000001.1
Acinetobacter johnsonii	NCTC10308^{T}	NZ_UFRV01000006.1
Acinetobacter kookii	ANC 4667	NZ_FMYO01000020.1
Acinetobacter kyonggiensis	ANC 5109	NZ_FNPK01000071.1
Acinetobacter lactucae	NRRL B-41902^{T}	NZ_LRPE00000000.1
Acinetobacter larvae	BRTC-1^{T}	NZ_CP016895.1
Acinetobacter lwoffii	NCTC5866^{T}	NZ_CAADHN010000002.1
Acinetobacter marinus	ANC 3699	NZ_FMYK01000020.1
Acinetobacter nectaris	CIP 110549^{T}	NZ_KI530712.1
Acinetobacter nosocomialis	LMG 10619^{T}	NZ_BBSR00000000.1
Acinetobacter oleivorans	DR1^{T}	NC_014259.1
Acinetobacter parvus	DSM 16617^{T}	NZ_KB849207.1
Acinetobacter piscicola	LW15^{T}	NZ_NIFO01000001.1
Acinetobacter pittii	CIP 70.29^{T}	NZ_APQP00000000.1
Acinetobacter populi	PBJ7^{T}	NZ_NIFO01000001.1
Acinetobacter pragensis	ANC 4149^{T}	NZ_LUAW01000001.1
Acinetobacter proteolyticus	NIPH 809^{T}	NZ_APOI00000000.1
Acinetobacter pseudolwoffii	ANC 5044^{T}	NZ_PHRG01000001.1
Acinetobacter puyangensis	ANC 4466	NZ_OANT01000035.1
Acinetobacter qingfengensis	CCUG 69710^{T}	NZ_VXKN00000000.1
Acinetobacter radioresistens	NBRC 102413^{T}	NZ_AP019740.1
Acinetobacter rudis	DSM 24031^{T}	NZ_BBRX01000151.1
Acinetobacter schindleri	CIP 107287^{T}	NZ_KB849571.1
Acinetobacter seifertii	NIPH 973^{T}	NZ_KB851195.1
Acinetobacter soli	KCTC 22184^{T}	NZ_BBNM00000000.1
Acinetobacter tandoii	DSM 14970^{T}	NZ_JHZG01000001.1
Acinetobacter tjernbergiae	DSM 14971^{T}	NZ_KB894353.1
Acinetobacter towneri	DSM 14962^{T}	NZ_KB849660.1
Acinetobacter ursingii	CIP 107286^{T}	NZ_APQA00000000.1
Acinetobacter variabilis	NIPH 2171^{T}	NZ_KB850111.1
Acinetobacter venetianus	RAG-1^{T}	NZ_AKIQ01000001.1
Acinetobacter wuhouensis	WCHA60^{T}	NZ_CP031716.1
Acinetobacter geminorum	J00019^{T}	JABELE000000000
Acinetobacter geminorum	J00460	n/a
